# DKA in a toddler negative to type 1 diabetes autoantibodies: unusual presentation of Kearn–Sayre syndrome

**DOI:** 10.1007/s00592-025-02509-z

**Published:** 2025-04-17

**Authors:** Annalisa Deodati, Novella Rapini, Diego Martinelli, Mafalda Mucciolo, Rosalba Carozzo, Riccardo Schiaffini, Stefano Cianfarani, Fabrizio Barbetti

**Affiliations:** 1 Endocrinology and Diabetes Unit, Bambino Gesù Children’s Hospital, IRCCS, Piazza S. Onofrio 4, 00163 Rome, Italy; 2 Department of Systems Medicine, University of Rome Tor Vergata, Rome, Italy; 3 Monogenic Diabetes Clinic, Endocrinology and Diabetes Unit, Bambino Gesù Children’s Hospital, IRCCS, Piazza S. Onofrio 4, 00163 Rome, Italy; 4 Division of Metabolic Diseases and Hepatology, Bambino Gesù Children’s Hospital, IRCCS, Rome, Italy; 5 Laboratory of Medical Genetics, Translational Cytogenomics Research Unit, Bambino Gesù Children’s Hospital, IRCCS, Rome, Italy; 6 Unit of Cell Biology and Diagnosis of Mitochondrial Disorders, Laboratory of Medical Genetics, Bambino Gesù Children’s Hospital, IRCCS, Rome, Italy; 7 Department of Women’s and Children Health, Karolinska Institute and University Hospital, Solna, Stockholm, Sweden

**Keywords:** DKA, GCK, Monogenic diabetes, Kearns–Sayre syndrome

The most frequent form of diabetes mellitus in the pediatric setting is Type 1 Diabetes (T1D). In children negative to T1D-related autoantibodies however, a defect in a single gene (Monogenic Diabetes, MD) is the likely cause of hyperglycemia [1]. MD subtypes are many but most patients bear defects in four of the Maturity Onset Diabetes of the Young (MODY) genes: *GCK*, *HNF1A*, *HNF4A*, *HNF1B* (1). Here we report a case with early onset diabetes initially categorized as T1D. Over 24 months after diabetes outset the unfolding of a complex phenotype pointed towards a different etiologic diagnosis.

The patient, a female, was born to non consanguineous parents after an uneventful pregnancy (birth weight: 3150 g, gestational age: 41 weeks). In the first two years of life she showed mild neurodevelopment delay (first words at 2 years). At the age of 3 y, 3/12 she was admitted for diabetic ketoacidosis (DKA) (plasma glucose 477 mg/dL, arterial pH 7.15). C-peptide was 0.2 ng/dL; the search for T1D autoantibodies (GAD, IA-2) and celiac disease antibodies was negative and she presented HLA-DQ2 genotype; family history for diabetes was mute. She was started on subcutaneous insulin at the dose of 0.4 UI/kg/d. A second T1D autoantibodies assay including IAA, ZnT8A, GADA and IA-2 A was again negative, likely excluding autoimmune diabetes.

Because spontaneous pathogenic variants in some MODY genes can lead to insulin requiring diabetes and can be easily misdiagnosed for T1D [2], the patient underwent a screening of *ABCC8*, *APPL1*, *GATA4*, *GATA6*, *GCK*, *HNF1A*, *HNF1B*, *HNF4A*, *INS*, *KCNJ11*, *NEUROD1*, *PDX1* and *RFX6* genes by next generation sequencing (NGS). NGS identified the heterozygous *GCK* variant p.Glu279Gly, classified in ClinVar (https://www.ncbi.nlm.nih.gov/clinvar/?term=GCK%5Bgene%5D&redir=gene) as of uncertain significance (VUS). In addition, *GCK*-MODY clinical phenotype is characterized by non progressive, mild fasting hyperglycemia and no need of pharmacological therapy [1]. Therefore, we concluded that the *GCK* variant was not the cause of diabetes in this patient (Rapini N, Patera PI, Schiaffini R, et al. *Acta Diabetol* 2023; 60:61–70) and should be regarded as incidental finding.

Six months after diabetes onset (age: 3 y, 9/12) an ECG revealed an atrioventrcular conduction delay, and 3 months later (age: 4 y) a pacemaker was implanted because of third degree atrio-ventricular block diagnosed after a lipothymic episode. Around the same time mild eyelid apoptosis was noted.

After 1 year from the diagnosis (4 y and 3/12), the child was still treated with a basal-bolus therapy using Lantus and Humalog before main meals, with a low insulin requirement (0.5 U/kg/d). HbA1c was 57 mmol/mol, peptide c 0.13 ng/mL. The child has followed a carbohydrate-balanced diet, although the parents reported hyporexia and food selectivity. The glycemic profile showed a fluctuating trend in blood glucose levels with frequent hypoglycemia episodes despite the low insulin requirement.

Given the poor glycemic control, at the age of 6 y, therapy with an insulin pump was initiated.

One year later (5 y of age) a sharp decrease of growth rate led to growth hormone stimulation tests that elicited normal results. Bone age was adequate for chronological age and other common causes of growth retardation (e.g. coeliac disease) were ruled out. At the age of 7 y retinal degeneration, worsening of lid ptosis and neurosensorial hearing loss were observed. The combination of neurosensorial deafness, non diabetic retinopathy, growth retardation, complete AV block and diabetes pointed towards a clinical diagnosis of Kearns–Sayre syndrome (KSS) [3, 4]. This in turn prompted the analysis of mitochondrial DNA by long-range PCR that revealed a large deletion of 7409 base pairs frequently found in KSS. Diagnosis of KSS dictated the re-evaluation of the endocrinological status of the patient, that revealed a progressively decreasing intact PTH (14.1→5.19→3.81, ref. values:15–65 pg/mL) and low Ca^++^ levels of 1.05 mmol/L (ref. values 1.16–1.32) (Table 1), leading to the diagnosis of primary hypoparathyroism. The function of thyroid, adrenal and pituitary is normal at the time of writing.

**Table 1:** Additional metabolic/hormonal features of present case not usually found in type 1 diabetes or common monogenic diabetes defects

	Lactate	NEFA/triglycerides	Ca^++^	Ca	*P*	PTH (intact)
Reference values	0.5–1.4 mmol/L	100–450 µM/L/<100 mg/dL	1.16–1.32 mmol/L	8.8–10.8 mg/dL	3.1–5–5 mg/dL	15–65 pg/mL
Patient values (Feb 2023)	3.75	n.a/74	0.98	8	4.9	31.2
Patient values (May 2023)	3.7	1252/71	1.05	8.3	5.7	14.1
Patient values (Oct 2023)	2.2	404/70	1.17 (Ca therapy)	9.1	4.5	5.19
Patient values (Apr 2024)	2	782/69	1.26 (Ca therapy)	10.3	3.7	3.81
Patient values (Sep 2024)	2.1	1158/110	1.61 (Ca therapy)	11.4	3.5	7.71

After the diagnosis, the dietary plan was adjusted by fixed carbohydrate amounts for main meals and snacks, with adjustment of pre-meal boluses. The family was educated on managing hypoglycemia and, most importantly, on managing insulin pump therapy during intercurrent episodes.

KSS is a rare neurodegenerative condition (1–9:100,000 live births) characterized by a progressive multisystemic disorder defined by the triad of pigmentary retinopathy, external ophthalmoplegia, and onset before the age of 20 years, with 1 or more additional features including cardiac conduction block, cerebrospinal fluid protein concentration > 100 mg/dL, or cerebellar ataxia. In addition, in about one third of KSS patients high serum lactate can be found (Table 1). Endocrine dysfunctions such as growth failure, hypoparathyroidism and hypogonadotropic hypogonadism are frequently seen in KSS (ranging between 35 and 67% of individuals with large deletions of mitochondrial DNA) [4]. Notably, it has been reported that about 10–15% of patients with KSS develop type 1-like diabetes but not during toddlerhood and very rarely as the presenting feature [4]. Thus the search for a genetic defect in K_ATP_ (*KCNJ11*, *ABCC8*) or *INS* genes was justified, but the severe, non syndromic diabetes initially observed in our proband was not compatible with a loss-of-function *GCK* variant. However, we cannot exclude that *GCK* mutation could have contributed to the apparent “anticipation” of diabetes onset in our patient [5].

In conclusion, in the presence of diabetes onset with negative antibodies and negative or not-correlated to the phenotype genetic result, as in our case, it is important to follow the patient over time to promptly identify the emergence of new clinical features that may help in achieving a correct diagnosis.
